# 한국 가정전문간호사의 욕창-실금관련피부염 시각적 감별 능력 영향요인: 횡단적 연구

**DOI:** 10.7586/jkbns.25.058

**Published:** 2025-11-18

**Authors:** Sun-Young Choi, Seungmi Park

**Affiliations:** 1 Department of Nursing, Chungbuk National University Hospital, Cheongju, Korea; 1 충북대학교병원; 2 Department of Nursing Science & Research Institute of Nursing Science, College of Nursing, Chungbuk National University, Cheongju, Korea; 2 충북대학교 간호학과

**Keywords:** Home care services, Pressure ulcer, Urinary incontinence, Fecal incontinence, Dermatitis, 가정간호, 욕창, 요실금, 변실금, 피부염

## Abstract

**Purpose:**

This descriptive survey aimed to identify factors influencing home health care advanced practice nurses’ ability to visually differentiate between pressure injuries (PIs) and incontinence-associated dermatitis (IAD).

**Methods:**

The study included 157 home health care advanced practice nurses currently employed at medical institutions nationwide. Data were collected from July 20 to November 5, 2024. Instruments consisted of a PI-IAD knowledge assessment tool (19 items) and a PI-IAD visual differentiation evaluation tool (21 images and related information).

**Results:**

Participants achieved an average score of 14.86 ± 2.06 out of 19 on the PI-IAD knowledge scale. The mean score for visual differentiation ability was 13.20 ± 4.15 out of 21. A significant positive correlation was found between PI-IAD knowledge and visual differentiation ability (r = .52, *p* < .001). Multiple regression analysis identified PI-IAD knowledge, age under 40 years, clinical experience of 10~20, 20~30, or over 30 years, and employment in clinics or general hospitals as significant predictors of visual differentiation ability. These variables explained 39% of the variance (F = 8.81, *p* < .001).

**Conclusion:**

Home health care advanced practice nurses demonstrated difficulty visually distinguishing between pressure injury stages and IAD. Therefore, practice-based educational programs are needed to improve anatomical understanding and visual discrimination skills.

## 서론

### 1. 연구의 필요성

욕창(pressure injury)은 주로 뼈 돌출부위에 장기간 압력이나 전단력이 가해짐으로써 피부와 그 밑의 조직에 국소적인 손상이 생기는 상태를 의미하며[1], 이는 대상자와 돌봄 제공자의 삶의 질을 저하시킬 뿐만 아니라, 재원 일수 및 의료비 부담을 증가시킨다[2]. 욕창이 발생하면 환자는 통증과 불편함을 겪고, 감염이나 패혈증 등 합병증 발생 위험이 증가하며, 이는 생명을 위협하는 요인을 초래하기도 한다[3]. 욕창의 정확한 사정 및 조기 발견, 그리고 효율적 간호 수행을 위해서는 간호사의 욕창에 대한 지식과 정확한 욕창 사정 능력이 중요하다[4]. 욕창 간호지식은 욕창분류체계에 따른 욕창 사정, 욕창 예방 및 관리, 통증 사정 및 관리, 상처 간호 등과 관련된 지식을 의미한다[5]. 욕창의 분류는 욕창 간호 사정에서 중요한 요소로 욕창분류체계는 욕창의 심각성 여부를 결정하는 기준이 된다[4].

가정간호는 일상생활에 도움이 필요한 만성질환자 등을 대상으로, 가정전문간호사가 가정을 직접 방문하여 기본 간호 제공 및 투약, 응급처치 등 다양한 간호서비스를 제공하는 것이다[6]. 가정간호는 지역사회 통합돌봄의 중요한 부분으로, 가정간호에 대한 수요는 앞으로도 더욱 증가할 것으로 예측된다. 가정전문간호사는 활력 징후 측정, 건강 상태 관찰, 수액 관리 및 정맥주사, 비위관 교환 및 관리, 정체 도뇨관 교환 및 간호 등의 기본간호 외에도 욕창 간호와 같은 다양한 간호업무를 수행한다[7]. 욕창 간호는 가정전문간호사의 주요 업무 중 하나로, 환자의 피부 상태를 정확하게 사정하고, 예방적 간호 및 치료적 중재를 제공하는 데 중요한 역할을 한다. 가정전문간호사는 환자의 가정에서 다른 의료진의 도움 없이 독립적으로 치료행위와 간호를 수행해야 하므로, 욕창을 정확히 사정하고 그에 맞는 간호를 제공할 수 있는 전문 지식과 기술을 갖추는 것이 매우 중요하다[8].

장기적인 와상환자는 욕창 외에도 실금관련피부염(incontinence-associated dermatitis, IAD)이 자주 발생한다. IAD는 실금으로 인해 피부가 소변이나 대변에 노출되어 발생하는 염증성 피부 질환이며[9], 특히 회음부를 포함한 골반 부위는 욕창의 주요 호발 부위로서 실금은 욕창의 발생과 밀접한 관련이 있다[10]. 실금으로 인해 피부가 소변이나 대변에 노출되면서 염증이 발생하면, 피부가 압력 등 외부자극에 취약해져 욕창으로 쉽게 진행이 될 수 있다[9,11]. 욕창과 IAD는 2000년 이후부터 구분하여 평가하고 있으나[12], 발생 부위가 유사하여 구분에 어려움이 있다. 이로 인해 IAD를 욕창으로 잘못 인식하여 부적절한 간호가 이루어지기도 한다[10,11]. 따라서 발생 원인에 따라 적절한 관리 접근법을 선택하기 위해서 간호사에게 정확한 욕창 단계 구분과 IAD를 구별할 수 있는 시각적 감별 능력이 필요하다.

시각적 감별 능력이란, 간호사가 욕창분류체계를 기준으로 욕창의 유무와 단계를 판단하고, IAD와 욕창을 시진을 통해 정확히 구별할 수 있는 능력을 의미한다. 특히, 욕창 사정은 피부 손상의 위치, 깊이, 조직 상태 등을 시진을 통해 체계적으로 관찰함으로써 욕창의 유무 및 단계를 평가하는 과정으로, 이는 효과적인 욕창 예방과 적절한 간호 중재를 위한 출발점이 되며, 간호사의 정확한 시진 능력은 필수적인 역량이다[13]. 현재까지의 욕창-IAD에 대한 지식과 시각적 감별 능력에 관한 국내 선행연구는 임상 간호사[14], 병원 간호사[15], 졸업 학년 간호 대학생[16], 방문 간호 요양 요원[17] 등을 대상으로 이루어졌다. 그러나, 선행연구를 살펴보면 욕창 지식과 시각적 감별 능력 수준은 높지 않으며[14-17], 욕창 분류에 새롭게 추가된 심부조직손상단계에 대한 시각적 감별 능력 또한 매우 낮은 수준을 보였고[14], 간호사의 IAD에 대한 지식 정답률도 임상실무에서 요구하는 정확성을 충족하기에 미흡하다는 보고가 있었다[18]. 그러나 가정전문간호사는 가정 내에서 욕창 간호를 수행할 빈도가 높고, 만성질환자 및 욕창 고위험군을 대상으로 독립적인 간호를 제공함에도 불구하고, 이들을 대상으로 욕창-IAD에 대한 지식과 시각적 감별 능력에 대해 살펴본 연구는 없었다. 따라서 본 연구는 가정전문간호사를 대상으로 욕창-IAD에 대한 지식과 시각적 감별 능력을 확인하고, 시각적 감별 능력의 영향요인을 파악함으로써 가정간호 실무 현장에서의 정확한 피부 상태 사정과 적절한 간호 중재를 위한 기초 자료를 제공하고자 한다.

### 2. 연구의 목적

본 연구의 목적은 가정전문간호사를 대상으로 욕창-IAD에 대한 시각적 감별 능력을 파악하기 위함이며, 구체적인 목적은 다음과 같다.

• 대상자의 욕창-IAD에 대한 지식과 시각적 감별 능력의 정도를 파악한다.

• 대상자의 일반적 특성에 따른 욕창-IAD에 대한 지식과 시각적 감별 능력의 차이를 파악한다.

• 대상자의 욕창-IAD에 대한 지식과 시각적 감별 능력의 상관관계를 파악한다.

• 대상자의 시각적 감별 능력의 영향요인을 파악한다.

## 연구 방법

### 1. 연구 설계

본 연구는 가정전문간호사의 욕창-IAD에 대한 지식과 시각적 감별 능력을 확인하고 시각적 감별 능력에 영향요인을 파악하기 위한 서술적 조사연구이다.

### 2. 연구 대상

본 연구의 대상자는 전국의 가정간호 서비스 의료기관에 소속된 가정전문간호사를 대상으로 수행하였으며, 연구의 목적을 이해하고 자발적으로 연구 참여에 동의한 자이다. 연구 대상자 수는 G*power 3.1.9.7 프로그램[19]을 이용하여 산출결과, 욕창 지식에 대한 선행연구[20,21]를 참고하여 효과 크기 0.15로 설정, 유의수준 .05, 검정력 .80을 설정하였다. 예측변수는 지식과 일반적 특성이 포함되었으며, 일반적 특성 중 유의한 차이를 보인 변수는 가변수(dummy variables)로 변환할 것을 고려하여 총 20개를 포함하였다. 다중회귀분석(multiple regression analysis)에서 필요한 최소 표본크기 산출결과, 157명이였으며 탈락률 약20%를 고려하여 최종 표본크기를 196명으로 설정하였다. 최종적으로 연구 참여에 동의한 199명의 설문지 중 응답이 불충분한 42명을 제외하고, 총 157명의 설문지를 자료 분석에 활용하였다.

### 3. 연구 도구

#### 1) 일반적 특성

대상자 일반적 특성은 연령, 성별, 최종 학력, 의료기관 유형, 임상 경력, 가정전문간호사 근무 경력, 욕창 간호 빈도, IAD 간호 빈도로 구성하였다.

#### 2) 욕창-IAD 지식

대상자의 욕창-IAD 지식을 측정하기 위한 도구로 선행연구와 욕창분류체계 및 IAD에 관한 문헌을 바탕으로 Lee 등[14]이 사용한 19개 항목의 도구로 측정하였다. 단계별 욕창-IAD에 대한 설명을 읽고, 각 문항의 내용이 옳으면 ‘예’, 틀리면 ‘아니요’라고 응답하도록 하였으며, 정답은 1점, 오답은 0점으로 환산하였다. 총 점수의 범위는 0~19점이며 점수가 높을수록 지식의 정도가 높음을 의미한다. 개발 당시의 신뢰도는 KR-20 (Kuder-Richardson Formula 20) =.76, 내용 타당도는 Content Validity Index (CVI) =.80이었다. 본 연구에서 신뢰도 KR-20 값은 .63이었다.

#### 3) 욕창-IAD 시각적 감별 능력

대상자의 욕창-IAD에 대한 시각적 감별 능력을 측정하기 위해 Lee 등[13]이 개발한 도구로, 21장의 사진과 함께 의학적 진단, 환자 기동성, 배변 상태, 경관 유동식 주입 여부, 상처 보유기간 등 환자의 임상적 상태 정보가 명시되어 있다. 21장의 사진은 6단계의 욕창 단계별 사진 16장, 창백성 홍반(blanching erythema) 사진 2장, IAD 사진 3장으로 구성하였다. 정답은 1점, 오답은 0점으로 환산하여, 점수의 범위는 0~21점, 점수가 높을수록 욕창분류체계 및 실금 관련 피부염의 시각적 감별 능력이 높음을 의미한다. 개발 당시의 신뢰도는 Multi-rater kappa=.81 (*p* < .001)이었고, 내용 타당도는 CVI =.83이었다. 본 연구에서 신뢰도 KR-20 값은 .78이었다.

### 4. 자료 수집

자료 수집은 2024년 7월 20일부터 11월 5일까지 진행되었으며, 전국 가정간호 서비스 의료기관에 근무중인 가정전문간호사를 대상으로 하였다. 자료 수집은 온라인 설문조사 방식으로 이루어졌으며, 구글폼(Google Forms, Google LL, Mountain View, California, USA)을 활용하였다. 설문지는 대한간호협회 산하 가정간호사회 협조를 받아 각 가정간호 서비스 의료기관에 배포되었고, 연구자가 직접 각 기관에 연락하여 연구의 목적과 내용을 설명한 후 설문지 URL을 전달하였다. 설문조사 첫 화면에는 연구의 목적, 방법, 개인 정보 보호, 익명성 및 비밀 보장, 연구 참여의 자발성, 참여 철회 가능성, 그리고 참여에 따른 혜택들이 명시되었으며, 연구에 동의한 대상자만 설문에 응답할 수 있도록 구성하였다. 또한 설문 참여 도중 의사가 철회된 경우, 인터넷 창을 종료하면 설문이 자동 종료되고, 저장되지 않도록 설정하였으며, 설문 응답에는 약 15~20분이 소요되었다.

### 5. 자료 분석

수집된 자료는 SPSS version 27.0 (IBM Corp., Armonk, NY, USA) 통계 프로그램을 이용하여 분석하였다. 대상자의 욕창-IAD 지식과 시각적 감별 능력에 대한 정도는 평균과 표준편차를 사용한 기술통계로 분석하였다. 일반적 특성에 따른 욕창-IAD 지식과 시각적 감별 능력의 차이에 대한 평가는 independent t-test, one-way ANOVA를 이용하고, 사후검정은 Scheffé’s test로 분석하였다. 욕창-IAD에 대한 지식과 시각적 감별능력 간의 상관관계는 Pearson correlation coefficient를 이용하여 분석하였다. 시각적 감별 능력 영향요인은 입력(enter)방법을 이용하여 다중 회귀 분석(multiple regression analysis)을 실시하였다.

### 6. 윤리적 고려

본 연구는 충북대학교 생명윤리심의위원회(IRB No. CBNU-2024-A-0018)의 심의를 거쳐 승인을 받은 후 연구를 수행하였다. 대상자에게 연구 참여 전 연구의 목적, 내용, 개인정보 보호, 익명성과 비밀 보장, 자발적 참여 및 철회 가능성 등에 대해 충분히 설명하였으며, 온라인 설문 첫 화면에서 동의 절차를 거친 후 설문에 참여할 수 있도록 하였다. 수집된 모든 자료는 컴퓨터를 이용해 코드화한 후 비밀번호가 설정된 파일에 안전하게 저장하였으며, 연구자 외에는 접근이 불가능하도록 하였다. 또한 연구와 관련된 모든 자료는 「개인정보보호법」 등 관련 법률에 따라 연구 종료 시점부터 3년간 보관 후, 영구적으로 삭제하여 복원이 불가능하도록 폐기할 예정이다.

## 연구 결과

### 1. 대상자의 일반적 특성

대상자의 평균 연령은 48.75 ± 10.57세였으며, 연령 분포는 50세 이상~60세 미만이 57명(36.0%)으로 가장 많았다. 성별은 여성이 156명(99.4%)로 대부분을 차지하였다. 최종 학력은 석사 학위 이상이 103명(65.6%)으로 높은 비율을 보였으며, 근무 중인 의료기관 유형은 종합병원이 58명(37.4%)으로 가장 많았다. 총 임상 경력은 평균 21.02 ± 9.39년이었으며, 20년 이상 30년 미만이 56명(35.7%)으로 가장 많았다. 가정간호 근무 경력은 평균 8.85 ± 8.71년으로 나타났고, 5년 미만이 68명(43.3%)으로 가장 높은 비율을 보였다. 욕창 간호 빈도는 주 1회 전후가 85명(54.8%)으로 가장 많았고, IAD 간호 빈도는 월 1회 이상이 65명(48.1%)으로 가장 높았다(Table 1).

### 2. 대상자의 욕창-IAD 지식과 시각적 감별 능력의 정도

**Figure 1.**
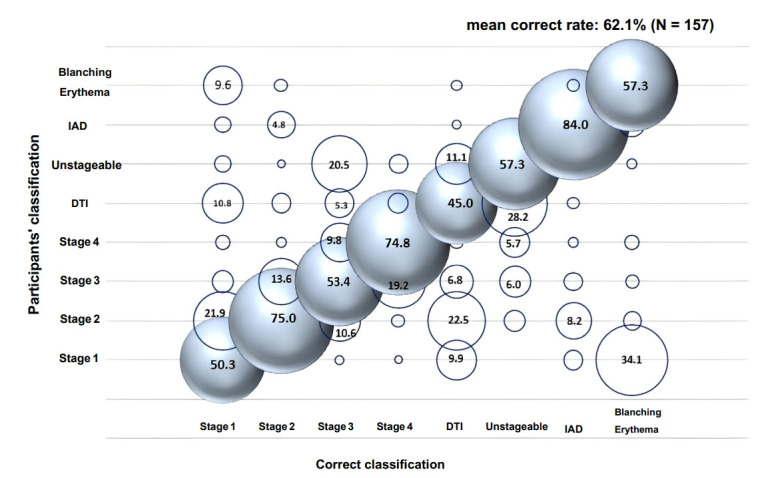
Correct classification rate for visual differentiation ability. IAD = Incontinence-associated dermatitis; DTI = Deep tissue injury.

대상자의 욕창-IAD에 대한 지식수준은 총 19점 만점에 평균 14.86점 ± 2.06점으로, 전체 평균 정답률은 78.2%였다(Table 2). 정답률이 가장 낮은 문항은 ‘욕창 3단계는 피하조직과 근막이 손상된 상태이다’로 정답률 19.7%였다. 대상자의 욕창-IAD에 대한 시각적 감별 능력의 수준은 임상 정보를 포함한 사진을 활용하여 측정하였으며, 총 21점 만점에 13.20 ± 4.15점으로, 전체 평균 정답률은 62.1%였다(Figure 1). 각 단계별로는 IAD가 84.0%로 가장 높은 정답률을 보였으며, 욕창 2단계(75.0%)와 욕창 4단계(74.8%)의 순으로 나타났다. 반면, 심조직손상(45%), 욕창 1단계(50.3%), 욕창 3단계(53.4%), 미분류 욕창 및 창백성 홍반(57.3%)은 비교적 낮은 정답률을 보였다. 욕창-IAD의 시각적 감별과정에서 오답률을 분석한 결과, 대상자의 34.1%가 창백성 홍반을 욕창 1단계로, 22.5%가 심부조직손상을 욕창 2단계로 잘못 분류하였으며, 욕창 1단계는 21.9%가 욕창 2단계로, 미분류 욕창은 28.2%가 심부조직손상으로 잘못 분류하였다.

### 3. 대상자의 일반적 특성에 따른 욕창-IAD 지식 차이

대상자의 일반적 특성에 따른 욕창-IAD에 대한 지식차이를 분석한 결과, 최종 학력(t = −2.00, *p* = .046), 총 임상 경력(F = 5.79, *p* < .001), 욕창 간호 빈도(F = 10.15, *p* < .001)에 따라 통계적으로 유의한 차이가 나타났다. 석사 이상의 학위를 가진 간호사(15.10 ± 2.03)가 학사 학위 소지자(14.41 ± 2.06)보다 더 높은 지식 점수를 보였고, 대학병원 근무자(15.61 ± 1.90)의 욕창-IAD 지식점수가 가장 높았다.

욕창 간호 빈도에 따른 지식 점수는 매일 수행하는 경우(15.44 ± 3.17)가 가장 높은 점수를 보였고, 사후 검정 결과, ‘거의 없음~월 1회 이상 수행하는 경우’에 비해 유의하게 높은 지식 점수를 보였다. 반면, 연령(t = 1.08, *p* = .360), 총 임상 경력(F = 2.56, *p* = .057), 가정 간호 경력(F = 0.20, *p* = .897), IAD 간호 빈도(F = 0.14, *p* = .870)에서는 통계적으로 유의한 차이가 없었다(Table 1).

### 4. 대상자의 일반적 특성에 따른 욕창-IAD 시각적 감별 능력 차이

대상자의 일반적 특성에 따른 욕창-IAD의 시각적 감별 능력 차이를 분석한 결과, 연령(F = 8.93, *p* < .001), 최종 학력(t = −2.25, *p* = .027), 의료기관 유형(F = 8.86, *p* < .001), 총 임상 경력(F = 6.54, *p* < .001), 욕창 간호 빈도(F = 10.16, *p* < .001)에 따라 통계적으로 유의한 차이가 나타났다. 시각적 감별 능력 점수는 40~49세(15.05 ± 3.44)에서 가장 높았으며, 사후 검정 결과 60세 이상(10.31 ± 3.53)의 연령대 보다 40~49세(15.05 ± 3.44) 연령대의 시각적 감별 능력 점수가 유의하게 높은 것으로 나타났다. 석사 이상의 학위를 가진 간호사(13.77 ± 3.72)가 학사 학위 소지자(12.11 ± 4.70)보다 더 높은 시각적 감별 능력 점수를 보였다. 의료기관 유형별 시각적 감별 능력 점수는 대학병원(14.37 ± 3.32)에서 근무하는 경우가 점수가 높았고, 사후 검정 결과, 병원에 비해 의원, 종합병원, 대학병원에서 근무자의 시각적 감별 능력 점수가 유의하게 높았다. 총 임상 경력 10~19년(14.26 ± 3.66)인 대상자의 욕창-IAD 시각적 감별 능력 점수가 가장 높았으며, 사후 검정 결과, 10년 미만 경력자 보다 20~29년 및 10~19년의 경력자의 점수가 유의하게 높았다. 욕창 간호 빈도에 따른 시각적 감별 능력 점수는 매일(15.44 ± 3.17) 수행하는 경우 가장 높았으며, 사후 검정 결과, 주 1회 전후 및 거의 없음~월 1회 수행하는 경우보다 유의하게 높은 시각적 감별 능력을 보였다. 반면, 가정간호 경력(F = 0.85, *p* = .469), IAD 간호 빈도(F =0 .62, *p* = .541)에서는 통계적으로 유의한 차이가 없는 것으로 나타났다(Table 1).

### 5. 가정전문간호사의 욕창-IAD 지식과 시각적 감별 능력 간의 상관관계

대상자의 욕창-IAD의 지식과 시각적 감별 능력 간의 상관관계를 분석한 결과, 두 변수 간에는 유의한 양의 상관관계 (r = .52, *p* < .001)가 있는 것으로 나타났다.

### 6. 대상자의 욕창-IAD 시각적 감별 능력 영향요인

대상자의 욕창-IAD의 지식 및 시각적 감별 능력 영향요인을 분석하기 위해 다중 회귀분석을 실시한 결과, 연령, 임상 경력, 최종 학력, 의료기관 유형, 욕창 간호 빈도, 욕창-IAD에 대한 지식이 유의한 상관성을 보여, 이들 변수를 독립변수로 설정하여, 가변수(dummy variable)로 변환하였으며, 입력(enter) 방법을 적용하여 회귀분석을 수행하였다. Durbin-Watson 통계량은 1.94으로, 2에 근접한 값을 보여 잔차의 독립성이 확보되었음을 확인하였고, 공차 한계(tolerance)는 0.87~0.95로 0.1 이상을 유지했으며, 분산 팽창 지수(variance inflation factor)는 1.05~1.16으로 10보다 낮아 독립변수 간 다중공선성의 문제는 없는 것으로 나타났다. 욕창-IAD에 대한 시각적 감별 능력의 회귀 모형은 통계적으로 유의하였고 (F = 8.81, *p* < .001), 욕창-IAD에 대한 지식(β = .40, *p* < .001), 연령 가변수 중 40세 미만(β = .30, *p* = .006), 총 임상경력 가변수 중 10년 이상 20년 미만(β = .24, *p* = .028), 20년 이상 30년 미만(β = .31, *p* = .022), 30년 이상(β = .26, *p* = .041), 의료기관 유형 가변수 중 의원(β = .22, *p* = .014) 및 종합병원(β = .19, *p* = .039)은 시각적 감별능력에 유의한 영향요인으로 나타났다. 해당 독립변수들은 시각적 감별능력의 40%를 설명하는 것으로 나타났다(Table 3).

## 논의

본 연구는 가정전문간호사를 대상으로 욕창-IAD에 대한 지식과 시각적 감별 능력의 정도를 확인하고, 시각적 감별 능력의 영향요인을 파악하고자 시행되었다. 가정전문간호사의 시각적 감별 능력에 영향을 미치는 가장 큰 요인은 욕창-IAD에 대한 지식이었다. 이 결과는 욕창에 대한 지식이 시각적 감별 능력에 중요한 영향요인으로 확인된 선행연구[15-17,22]의 결과와 일치하였다. 즉, 욕창에 대한 지식수준이 높을수록 시각적 감별 능력이 향상된다는 것을 의미한다.

한편, 본 연구에 참여한 가정전문간호사의 욕창-IAD에 대한 지식 점수 14.86점은 동일한 측정도구를 사용한 선행연구들과 비교할 때 상대적으로 높은 수준이었다. 구체적으로, 졸업학년 간호대학생을 대상으로 한 Cho 등[16]의 연구결과 11.89점, 방문 간호 요양요원을 대상으로 한 Kwon [17]의 연구결과 12.86점, 그리고 Park 등[15]의 연구결과 12.65점와 비교했을 때 본 연구 참여자의 평균점수는 높은 것으로 나타났다. 이는 본 연구 대상자가 석사과정의 교육수준을 자격요건으로 하는 전문간호사라는 특성 때문으로 생각된다. 그러나, 가정전문간호사의 욕창-IAD에 대한 지식 정답률은 78.2%로 전체 19개 문항 중 7개 문항의 정답률이 전체 평균 정답률(78.2%)보다 낮았다. 가장 낮은 정답률을 보인 문항은 ‘욕창 3단계는 피하조직과 근막이 손상된 상태이다‘로 정답률이 19.7%였다. 이 문항은 Kwon[17]의 연구에서 33.8%, Park 등[15]의 연구에서 10.9%, Lee와 Park [22]의 연구에서 22.8%의 정답률을 보이며 공통적으로 가장 낮은 정답률을 보인 문항으로 확인되었다. 이러한 결과는 근막을 포함한 근육의 손상 여부에 따라 욕창 3단계와 4단계를 구분해야 하는 점을 포함하여 욕창의 단계별 특징에 대한 교육의 필요성을 시사한다. 또한, ‘IAD의 경우 상처 기저부(wound bed)에 괴사조직이 관찰되지 않는다’는 문항도 정답률이 43.3%로 낮게 나타나 가정전문간호사들이 욕창과 IAD를 감별하는 데 혼란을 겪고 있음을 보여준다. 욕창은 산소요구도가 높은 심부조직이 뼈 돌출부 등에 의해 직접 압력을 받아 손상이 일어난 후 피부표면으로 나타나는(bottom to up) 경우가 많은 반면, IAD은 피부 자극 물질에 의한 피부표면의 부분층 손상을 유발하는 경우가 많다[23]. 두 상처의 구분에서는 피부손상의 발생 위치가 매우 중요하여, 욕창은 보통 뼈가 돌출한 부위에서 발생하고, 압박에 의해 조직이 눌려져서 허혈이 유발되는 외부의 힘이 존재하며, 그 손상이 일어나는 데에 조직을 짓누르는 것이 포함되지 않았으면 실금과 관련된 가능성이 더 높다[23].

욕창과 IAD의 구분은 상처 발생 원인을 이해하고 상처에 대한 중재 계획을 제공하는데 있어 매우 중요하므로, 가정전문간호사에게 이 두가지 상처 발생에 대한 병태생리학적 이해를 강조하는 실무교육이 필요하다. 향후 욕창-IAD 감별 능력 향상을 위한 교육 프로그램은 실제 사례기반 중심으로 욕창 3단계에 대한 정확한 정의 함께, 근막손상이 포함된 욕창 4단계의 차이를 구분할 수 있는 해부학적 지식의 적용을 강조해야 하며, IAD관련 특성에 대한 교육 강화가 필요함을 시사한다.

임상 정보를 포함한 사진을 활용하여 욕창 단계를 분류하는 시각적 감별능력 수준을 파악한 결과, 총 21점 중 평균 13.02점, 전체 평균 정답률은 62.1%로 나타났다. 이는 동일한 도구를 사용한 선행연구의 졸업 학년 간호대학생을 대상으로 한 Cho 등[16]의 연구(7.48점), 방문간호 요양 요원을 대상으로 한 Kwon [17]의 연구(7.78점), 병원간호사 대상으로 한 Park 등[15]의 연구(11.43점), Lee와 Park [22]의 연구(6.80점)보다 높은 수준이었다.

욕창과 IAD의 시각적 감별 능력 정답률에서는 심부조직손상 단계 정답률이 45.0% (71명)로 가장 낮게 나타났고, 대상자의 22.5%가 심부조직손상을 욕창 2단계로, 28.2%가 미분류 욕창을 심부조직손상으로 잘못 분류하였다. 이러한 결과는 Park 등[15], Cho 등[16], Kwon [17], Lee와 Park [22]의 선행연구 결과와도 일치한다. 이러한 오류 결과는 욕창-IAD 간의 시각적 감별 과정에서 욕창 병변의 깊이와 조직손상 정도를 시각적으로 정확히 판단하는데 어려움이 있음을 시사하며, 심부조직손상을 다른 욕창 단계와 오인하는 문제가 가장 빈번히 발생함을 나타낸다. 심부조직손상(deep tissue injury)은 압력이나 전단력에 의한 연한 조직손상으로 피부 탈색 또는 보라색, 갈색수포가 나타나는 손상이며, 욕창 2단계는 표피와 진피가 부분적으로 손상이 특징으로 얕은 궤양, 수포, 미란 등이 나타난다. 또한, 미분류욕창은 괴사조직으로 덮여 상처의 깊이를 알 수 없는 전층 피부 손상으로[5]. 심부조직손상의 표재성 병변과 괴사조직을 구분해 내는 것이 쉽지 않음을 나타내며, 이로 인해 시각적 평가만으로는 병변의 실제 깊이와 심각도를 정확히 판단하기 어려우므로, 임상 사례와 실제 환자 사례를 활용한 교육 프로그램이 필요하다[24].

본 연구 결과 대상자의 34.1%가 창백성 홍반을 욕창 1단계로 오인하였다. 창백성 홍반은 외부 압력으로 인해 피부의 모세혈관이 일시적으로 확장되어 발생하는 붉은 피부 반응이며. 압박 검사시 일시적으로 창백해졌다가 압력이 해제되면 원래의 색으로 회복되는 특성을 보인다. 반면, 욕창 1단계는 피부 손상이 없는 비창백성 홍반 상태로, 주로 뼈 돌출부위에 발생하며 눌러도 하얗게 되지 않는 발적 상태이다[2,24]. 이처럼 창백성 홍반(blanchable erythema)과 욕창 1단계(pressure injury stage 1)는 피부 표면에 비슷한 홍반성 병변이 관찰되며, 외형적으로 유사해 시각적 감별 오류가 발생한 것으로 해석된다.

이러한 결과를 바탕으로, 욕창의 단계별 병태생리 및 시각적 특징을 명확히 이해하는 것이 중요하며, 특히, 해부학적 구조와 병변의 진행 과정에 대한 이해를 높일 수 있는 지식 교육을 강화하고, 실제 가정간호 실무 현장에서 요구되는 시각적 감별능력을 향상시킬 수 있는 심화 교육과정의 개발이 필요하다고 판단된다. 최근에는 욕창 간호 교육 앱 및 AI 기반 욕창 관리 애플리케이션을 활용하여, 욕창 사정 및 분류 능력을 향상시키려는 연구들이 보고되고 있다[25-27]. 이러한 디지털 도구는 반복 학습과 시각적 피드백을 제공하여 간호사의 시각적 감별 능력을 효과적으로 개선하고, 욕창 환자 간호의 질적 향상에 기여할 수 있다. 따라서 향후 교육과정에서는 이론 및 실습교육과 함께 디지털 기반 학습 도구를 활용함으로써 간호사의 전문 역량을 보다 체계적이고 효과적으로 향상시킬 수 있을 것이다.

가정전문간호사의 욕창-IAD에 대한 시각적 감별 능력에 영향을 미치는 다른 요인으로는 연령, 총 임상경력, 의료기관 유형으로 나타났다. 본 연구의 연령에 대한 분석 결과, 60대에 비해 40세 미만의 연령층에서 유의하게 높은 점수를 나타냈으며, 연령이 낮을수록 시각적 감별 능력이 높은 것으로 나타났다. 반면, 총 임상경력은 10년 미만보다 10년 이상의 경력에서 시각적 감별능력이 높게 나타났으며, 임상 경력이 많을수록 시각적 감별능력이 높은 것으로 나타났다. 이러한 결과는 연령이 많다고 해서 반드시 감별 능력이 우수한 것은 아니며, 실질적인 임상 경험의 축적이 시각적 감별 능력 향상에 더 직접적인 영향을 줄 수 있음을 시사한다. 이는 Lee 등[14], Park 등[15]의 선행연구와 유사하며, 이들 연구에서도 젊은 연령층에서 상대적으로 높은 시각적 감별능력을 보였으나, 임상경력이 높은 집단에서도 감별능력이 우수하게 나타나 연령보다는 임상 경험의 질과 양이 더 중요한 요인임을 보여준다.

본 연구의 의료기관유형에 따른 분석 결과, 병원급 의료기관에 근무하는 가정전문간호사의 시각적 감별능력 점수가 의원, 종합병원, 대학병원에서 근무하는 가정전문간호사에 비해 유의하게 낮은 것으로 나타났다. 이러한 결과는 동일한 평가 도구를 사용한 선행연구인 Kwon [17]의 연구에서 시각적 감별능력이 의원, 병원, 종합병원 순으로 향상되었다는 결과와 다르다. 이와 같은 차이는 해당 기관에 근무하는 가정전문간호사의 임상 및 실무 경험차이에서 기인한 것으로 해석할 수 있다. 추가 분석을 실시한 결과 본 연구의 의원, 종합병원, 대학병원에 근무하는 가정전문간호사가 병원급 의료기관의 근무자 보다 임상경력, 가정간호 경력, 욕창과 IAD 간호 경험 및 간호 빈도가 높았다. 병원급 의료기관 근무자의 평균 임상경력은 19.24 ± 11.63년, 가정간호경력은 6.76 ± 9.01년으로 전체 대상자의 평균 임상경력(21.02 ± 9.39년), 가정간호경력(8.85 ± 8.71년) 보다 낮았고, 욕창-IAD 간호에 대한 경험과 간호 수행 빈도도 병원급 의료기관 근무자에서 낮았다. 즉, 병원급 의료기관에 근무하는 가정전문간호사가 다른 유형의 의료기관(의원, 종합병원, 대학병원) 근무자에 비해 총 임상경력뿐 아니라, 가정간호 및 욕창-IAD 관련 간호 수행 경험이 상대적으로 부족하였다. 따라서 시각적 감별능력의 차이는 의료기관의 규모나 유형보다는, 실제 가정간호 실무 현장에서 경험하는 상처 간호 경험과 빈도에 따라 더 밀접한 관련이 있음을 시사한다.

본 연구의 제한점으로는 전국 가정간호 서비스를 제공하는 의료기관에 소속된 가정전문간호사를 대상으로 수행되었으나, 실제 연구에 참여한 대상자의 수가 제한적이었기 때문에 연구 결과를 전체 가정전문간호사 집단에 일반화하는 데는 한계가 있을 수 있다. 또한, 욕창-IAD에 대한 지식은 자가 보고식 설문을 통해 지식을 측정하였기 때문에, 응답자의 주관적 판단에 따라 실제 욕창-IAD에 대한 지식 수준과 감별 능력을 정확히 반영하지 못했을 가능성도 존재한다. 아울러, 본 연구에서 사용한 욕창-IAD 지식 측정 도구의 신뢰도는 비교적 낮은 수준으로 나타났다. 해당 도구는 이분형 문항 형식으로 구성되어 있어 내적 신뢰도가 낮게 나타날 수 있다. 난이도•변별도 분석, 검사-재검사 신뢰도(test–retest reliability), 문항반응이론에 기반하여 신뢰도를 측정할 필요가 있다. 그럼에도 본 연구는 가정전문간호사의 욕창-IAD에 대한 지식과 시각적 감별 능력 간의 관련성을 규명하고, 실무적 역량 강화를 위한 기초자료를 제공하였다는 점에서 의의가 크다. 이러한 연구 결과는 향후 가정전문간호사의 전문성 향상과 교육 프로그램 개발에 있어 핵심적인 기초자료로 활용될 수 있을 것이다.

## 결론

본 연구는 가정전문간호사를 대상으로 욕창-IAD에 대한 시각적 감별 능력에 영향을 미치는 요인을 파악하기 위해 수행되었으며, 연구 결과, 욕창-IAD에 대한 지식 수준, 연령, 임상경력, 의료기관 유형이 시각적 감별 능력에 영향을 미치는 것으로 나타났고, 이들 요인은 시각적 감별능력의 40%를 설명하였다. 본 연구는 정확한 욕창 사정과 중재를 위한 기초자료를 제공함으로써, 가정전문간호사의 임상 실무 적용 및 교육 프로그램 개발에 실질적인 기반을 마련했다는 점에서 의의가 있다.

향후 연구에서는 보다 다양한 지역과 기관에 근무하는 가정전문간호사를 대상으로 표본의 대표성을 확보할 수 있는 연구 설계를 통해 연구 결과의 일반화 가능성을 높일 필요가 있다. 또한 욕창-IAD에 대한 지식과 시각적 감별 능력 향상을 위해 이론 교육뿐만 아니라, 실무 중심의 교육 방법을 포함하는 훈련 프로그램 개발이 요구된다. 아울러, 보다 객관적이고 다양한 측정 도구를 활용하여 실제 지식 수준과 시각적 감별 능력을 정확히 평가하는 후속연구가 필요하다.

## Figures and Tables

**Table 1.** Comparison of Scores for Pressure Injuries, the Incontinence-associated Dermatitis Knowledge Test, and Visual Differentiation Ability (N = 157)

Variables	Categories	n (%) or M ± SD	PI & IAD knowledge				PI & IAD visual differentiation ability		
			M ± SD		t or F (*p*)		M ± SD		t or F (*p*)
					Scheffé's test				Scheffé's test
Age (years)	< 40^a^	32 (20.0)	14.72 ± 2.28		1.08 (.360)		12.28 ± 4.77		8.93 (< .001) b > d
	40~49^b^	42 (27.0)	15.10 ± 1.86				15.05 ± 3.44		
	50~59^c^	57 (36.0)	15.04 ± 2.21				13.67 ± 3.74		
	≥ 60^d^	26 (17.0)	14.27 ± 1.73				10.31 ± 3.53		
		48.75 ± 10.57							
Sex	Women	156 (99.4)	-		-		-		-
	Men	1 (0.6)							
Education	Bachelor's degree	54 (34.4)	14.41 ± 2.06		−2.00 (.046)		12.11 ± 4.70		−2.25 (.027)
	Master's degree or higher	103 (65.6)	15.10 ± 2.03				13.77 ± 3.72		
Type of clinical institutions	Clinic^a^	23 (14.8)	15.04 ± 20.3		5.79 (< .001) b < d		13.35 ± 3.76		8.86 (< .001) b < a,c,d
	Hospital^b^	35 (22.6)	13.74 ± 2.16				10.29 ± 3.95		
	General hospital^c^	58 (37.4)	14.93 ± 1.89				14.07 ± 4.20		
	University hospital^d^	41 (25.2)	15.61 ± 1.90				14.37 ± 3.32		
Total clinical career (years)	< 10^a^	23 (14.6)	14.04 ± 2.08		2.56 (.057)		10.17 ± 4.48		6.54 (< .001) a < b,c
	10~19^b^	42 (26.8)	15.48 ± 1.82				14.26 ± 3.66		
	20~29^c^	56 (35.7)	14.79 ± 2.16				14.00 ± 3.79		
	≤ 30^d^	36 (22.9)	14.78 ± 2.03				12.64 ± 4.13		
		21.02 ± 9.39							
Home health care nurse career (years)	< 5^a^	68 (43.3)	14.81 ± 2.09		0.20 (.897)		12.69 ± 4.38		0.85 (.469)
	5~9^b^	33 (21.0)	14.73 ± 1.82				13.45 ± 3.85		
	10~14^c^	19 (12.1)	15.16 ± 2.24				14.32 ± 3.40		
	≤ 15^d^	37 (23.6)	14.92 ± 2.19				13.32 ± 4.33		
		8.85 ± 8.71							
Frequency of PI care^†^	Rarely or more than once a month^a^	45 (29.0)	11.31 ± 4.78		10.15 (< .001) a < c		11.31±4.78		10.16 (< .001) a <b < c
	Around once a week^b^	85 (54.8)	13.67 ± 3.49				13.67 ± 3.49		
	Daily^c^	25 (16.2)	15.44 ± 3.17				15.44 ± 3.17		
Frequency of IAD care^‡^	Rarely	36 (26.7)	14.80 ± 1.65		0.14 (.870)		13.44 ± 3.93		0.62 (.541)
	More than once a month	65 (48.1)	15.03 ± 2.08				12.93 ± 4.73		
	Around once a week-daily	34 (25.2)	14.91 ± 2.45				13.91 ± 3.32		
PI-IAD knowledge		14.86 ± 2.06		Range (0~19)		Min (10)	Max (19)	100 Point Scale (78.21)	
PI-IAD visual differentiation ability		13.20 ± 4.15		Range (0~21)		Min (1)	Max (21)	100 Point Scale (62.16)	

IAD = Incontinence-associated dermatitis, M = Mean; SD = Standard deviation; PI = Pressure injury. ^†^N = 155: Only participants with experience in PI care; ^‡^N = 135: Only participants with experience in IAD care.

**Table 2.** Correct Answer Rates for PI-IAD Knowledge (N = 157)

Items	Correct answer rate n (%)
1. A stage 1 pressure injury is characterized by the damaged skin area being over a bony prominence and exhibiting non-blanchable erythema (skin redness that does not turn white when pressed). (T)	147 (93.6)
2. If erythema is observed in the perineal skin damage area caused by fecal or urinary incontinence, it is considered a pressure injury even without pressure. (F)	114 (72.6)
3. An area of skin damage with blanchable erythema (skin that turns white when pressed) is not considered a pressure injury. (T)	94 (59.9)
4. Dermatitis caused by moisture, such as incontinence, is closely related to the subsequent development of pressure injuries. (T)	147 (93.6)
5. Pressure injuries are primarily caused by pressure or a combination of pressure and shear forces. (T)	155 (98.7)
6. The presence of a serous blister without skin damage on a pressure-exposed area indicates a stage 2 pressure injury. (T)	131 (83.4)
7. The nose, ears, occiput, and ankle areas have little to no subcutaneous fat anatomically, making it difficult for pressure injuries to progress to stage 3 in these regions. (F)	142 (90.4)
8. In cases of incontinence-associated dermatitis, necrotic tissue is not observed in the wound bed. (T)	68 (43.3)
9. If the heel is covered with dry eschar and no erythema is observed, it does not need to be removed. (T)	79 (50.3)
10. A suspected deep tissue injury pressure injury may progress to form black dry eschar, even if detected and treated early. (T)	137 (87.3)
11. If the wound area is covered with black dry eschar or yellow slough, obscuring the wound bed, it is classified as an unstageable pressure injury. (T)	143 (91.1)
12. Secondary skin infections, such as fungal infections, can occur in cases of incontinence-associated dermatitis. (T)	151 (96.2)
13. The incidence of incontinence-associated dermatitis is higher in cases of fecal incontinence than urinary incontinence. (T)	137 (87.3)
14. If a portion of the skin appears purple or maroon in color or there are blood-filled blisters, it indicates the stage of deep tissue injury. (T)	146 (93.0)
15. If the skin is damaged by moisture, such as from fecal incontinence, it is considered a stage 2 pressure injury, even if it is not located over a bony prominence. (F)	83 (52.9)
16. In stage 4 pressure injuries, muscle, tendon, or bone is exposed. (T)	154 (98.1)
17. Stage 3 and stage 4 pressure injuries may have necrotic tissue, undermining, tunneling, or dead space. (T)	154 (98.1)
18. Stage 3 pressure injuries involve damage to the subcutaneous tissue and fascia. (F)	31 (19.7)
19. If a stage 3 pressure injury heals and granulation tissue forms, it is classified as a stage 2 pressure injury. (F)	120 (76.4)
Mean correct answer rate	78.2%

PI = Pressure injury; IAD = Incontinence-associated dermatitis; T = True; F = False.

**Table 3.** Factors Affecting PI-IAD Visual Differentiation Ability (N = 157)

Variables		B	SE	β	t	*p*
(Constant)		−4.25	2.16		−1.96	.052
PI-IAD knowledge		0.78	0.14	.40	5.70	< .001
Age (years) (ref= ≥ 60)	< 40	2.75	0.99	.29	2.79	.006
	50~59	1.36	0.85	.16	1.60	.112
Total clinical career (years) (ref=<10)	10 ≤~< 20	2.23	1.00	.24	2.23	.028
	20 ≤~< 30	2.64	1.14	.31	2.31	.022
	≥ 30	2.53	1.23	.26	2.06	.041
Education (ref= bachelor's degree)	Master’s degree or higher	1.24	0.91	.11	1.37	.175
Type of clinical institutions (ref= hospital)	Clinic	1.87	0.75	.22	2.48	.014
	General hospital	1.75	0.84	.19	2.09	.039
	University hospital	0.04	0.61	.01	0.06	.953
Frequency of PI care (ref= rarely or more than once a month)	Around once a week-daily	1.16	0.89	.11	1.30	.196
R^2^ = .45, Adjusted R^2^ = .40, F = 8.80, *p* < .001, Durbin-Watson = 1.93						

PI = Pressure injury; IAD = Incontinence-associated dermatitis; ref = Reference; SE = Standard error.

## Data Availability

The data supporting this study's findings are accessible from the corresponding author upon reasonable request. Due to privacy and ethical considerations, the data are not publicly available.
